# Silencing of the Laccase (*lacc2*) Gene from *Pleurotus ostreatus* Causes Important Effects on the Formation of Toxocyst-like Structures and Fruiting Body

**DOI:** 10.3390/ijms24098143

**Published:** 2023-05-02

**Authors:** Anahí Armas-Tizapantzi, José Luis Martínez y Pérez, Francisco José Fernández, Gerardo Mata, Laura V. Hernández-Cuevas, Elvia Ortiz Ortiz, Edelmira García Nieto, Araceli Tomasini, Edgar Sierra-Palacios, Jaime Marcial-Quino, Alba Mónica Montiel-González

**Affiliations:** 1 Doctorado en Ciencias Biológicas, Centro Tlaxcala de Biología de la Conducta, Universidad Autónoma de Tlaxcala, Tlaxcala 90062, Mexico; anahiarmas@gmail.com; 2 Centro de Investigación en Genética y Ambiente, Universidad Autónoma de Tlaxcala, Tlaxcala 90120, Mexico; jlmarpe@hotmail.com (J.L.M.y.P.); fungicuevas@hotmail.com (L.V.H.-C.); mirosgn@yahoo.com.mx (E.G.N.); 3 Departamento de Biotecnología, CBS, Universidad Autónoma Metropolitana-Iztapalapa, Ciudad de Mexico 09340, Mexicoatc@xanum.uam.mx (A.T.); 4 Instituto de Ecología, A.C., Xalapa 91073, Mexico; gerardo.mata@inecol.mx; 5 Facultad de Odontología, Universidad Autónoma de Tlaxcala, Tlaxcala 90000, Mexico; elviaortizortiz@yahoo.com.mx; 6 Colegio de Ciencias y Humanidades, Plantel Casa Libertad, Universidad Autónoma de la Ciudad de México, Ciudad de Mexico 09620, Mexico; edgar.sierra@uacm.edu.mx

**Keywords:** gene expression, transformants, morphogenesis, RNAi, scanning electron microscopy, solid culture

## Abstract

A wide variety of biological functions, including those involved in the morphogenesis process of basidiomycete fungi, have been attributed to laccase enzymes. In this work, RNA interference (RNAi) was used to evaluate the role of the laccase (*lacc2*) gene of *Pleurotus ostreatus* PoB. Previously, transformant strains of *P. ostreatus* were obtained and according to their level of silencing they were classified as light (T7), medium (T21) or severe (T26 and T27). The attenuation of the *lacc2* gene in these transformants was determined by RT-PCR. Silencing of *lacc2* resulted in a decrease in laccase activity between 30 and 55%, which depended on the level of laccase expression achieved. The silenced strains (T21, T26, and T27) displayed a delay in the development of mycelium on potato dextrose agar (PDA) medium, whereas in the cultures grown on wheat straw, we found that these strains were incapable of producing aerial mycelium, primordia, and fruiting bodies. Scanning electron microscopy (SEM) showed the presence of toxocyst-like structures. The highest abundance of these structures was observed in the wild-type (PoB) and T7 strains. However, the abundance of toxocysts decreased in the T21 and T26 strains, and in T27 they were not detected. These results suggest that the presence and abundance of toxocyst-like structures are directly related to the development of fruiting bodies. Furthermore, our data confirm that *lacc2* is involved in the morphogenesis process of *P. ostreatus*.

## 1. Introduction

The genus *Pleurotus* comprises a group of edible mushrooms of biotechnological interest due to their nutritional value, medicinal properties, ecological functions, and environmental applications [1]. *Pleurotus ostreatus*, is one of the best known and cultivated species on a large scale worldwide [2]. Due to the high content of carbohydrates, fibers, minerals, amino acid vitamins, and proteins, this mushroom is considered a functional food, with nutritional and medicinal value [2,3]. Therefore, for edible and commercial purposes, its growth has been evaluated on different agro-industrial residues such as cereal straw, maize straw, sugarcane bagasse, wheat straw, olive residues, and wetland vegetations [2,4,5], which in turn has allowed the recycling of waste organic solids, the isolation of secondary metabolites [3], and the production of biofuels and bioproducts [6].

Furthermore, *P. ostreatus* secretes enzymes that have the ability to degrade organic matter, particularly cell wall components (cellulose, hemicellulose, and lignin) [7]. These enzymes, include lignin peroxidase (LiP), manganese peroxidase (MnP), versatile peroxidase (VP), and laccase (Lacc) [8]. Laccase is an enzyme involved in the degradation of lignin and other phenolic compounds [9]. Laccases are blue multicopper oxidase enzymes that contain four copper atoms, classified into three groups (T1, T2, and T3) according to their spectroscopic properties. The T1 type is responsible for the characteristic blue color of the enzyme [10,11]. These enzymes are considered versatile, because they have the capacity to catalyze a large number of reactions involving phenolic and non-phenolic compounds, using only O_2_ as the final electron acceptor and generating H_2_O [6]. Hence, it plays an important role in the treatment of environmental contaminants such as xenobiotic compounds and dyes [11,12].

On the other hand, the physiological participation of laccases in fungal growth is still not completely elucidated. However, some previous studies have described that laccases are involved in various biological events, such as pigmentation, virulence, pathogenesis, stress resistance, sporulation, and the formation of fruiting bodies [13,14]. Sun et al. [15] reported that there is a correlation between the production of laccase enzymes secreted during their growth stages in different edible mushrooms, including those of the genus *Pleurotus*. Genome sequencing has revealed that the number of laccase-coding genes varies between *P*. *ostreatus* strains. Transcriptomic studies have shown its differential expression during different stages of development. *P. ostreatus* cultivated in liquid and solid media constantly expresses *lacc2* and *lacc10* of the 12 genes present in its genome [16]. Pezella et al. [17], through a transcriptional analysis of the same fungus in different stages of development, reported that LACC10 plays an important role during its vegetative growth and lowers its expression at the beginning of the fungus fruiting. LACC2 is involved in the fruiting process. For this reason, it was of interest to analyze the regulation of the *lacc2* gene of *P. ostreatus* PoB through gene silencing, in order to evaluate the morphological implications on the fungus.

Within the field of functional genomics, RNA interference (RNAi) silencing has been considered a useful tool for the identification of gene function in a wide range of eukaryotic organisms, including fungi [18]. The functionality of RNAi on laccase genes has been demonstrated in different species of fungi. In *Lentinula edodes*, *lcc1* inhibition showed negative effects on the morphology of the mycelium [19], as well as on the structure of the cell wall [20]. Jin et al. [21] reported that in *Ganoderma tsugaela*, deletion of the *lacc1* gene affects pigmentation as well as stipe elongation during fruiting body development. In *Setosphaeria turcica*, inactivation of the *lacc2* gene causes cell wall thinning, and mutant strains are unable to produce conidia [22]. In the same fungus, *lacc6* attenuation did not affect growth or morphology, but did affect peroxisome function [23]. Thus, gene silencing has become a useful tool to analyze the regulation of specific genes, allowing for the manipulation of their expression and the evaluation of their biological functions. Another advantage offered by RNAi silencing is the ability to obtain strains with different levels of gene attenuation with the respective decrease in enzymatic activity, which allows for analysis of the effects on organisms, as previously demonstrated in *P. ostreatus* [24].

The aim of this work was to analyze transforming strains of *P. ostreatus* previously obtained by Armas-Tizapantzi et al. [24], with different levels of silencing of the *lacc2* gene. The purpose was to determine if this protein (LACC2) is directly related to the formation of fruiting bodies and toxocyst-like structures. Experiments were carried out in solid culture from which macroscopic and microscopic morphological determinations during its different stages of development were made.

## 2. Results

### 2.1. Evaluation of Laccase (lacc2) Gene Expression

**Figure 1:**
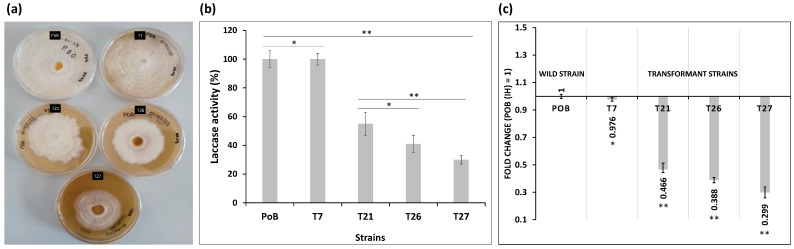
Analysis of *lacc2* gene silencing in *P. ostreatus* PoB. (**a**) Evaluation of growth and phenotype changes of the mycelium of PoB and transformants (T7, T21, T26, and T27) in PDA medium. (**b**) Determination of enzymatic activity with 2,6-dimetoxiphenol. (**c**) Measurement of the transcripts of all the strains grown on wheat straw for 38 days of culture. The actin gene was used as the reference gene. Results are presented as the mean ± standard deviation of three replicates. Statistical analysis was performed with one way ANOVA, followed by Tukey’s test (* *p* ≤ 0.05, ** *p* ≤ 0.01).

Previously obtained transformants [24] with a silenced *lacc2* gene were used in this work in order to evaluate their effect on the formation of macro- (fruiting body) and microscopic (toxocyst) structures of the fungus. Four transformants were analyzed in this work, with different levels of silencing, namely, T7 (light), T21 (medium), and T26 and T27 (severe). To verify that the transformants continue to preserve the silenced *lacc2* gene, different tests were performed on them. All strains were cultivated in Petri dishes containing PDA medium. After eight days, the growth of the wild-type (PoB) strain and the T7 transformant were similar. However, the growth of strains with medium (T21) and severe (T26 and T27) attenuation was delayed (Figure 1a). In these strains, the mycelium covered the entire medium after eighteen days. Even the phenotype of the mycelium was different, with a higher density in T21 and T26, whereas in T27, the mycelium was thin and transparent compared to the PoB control.

The laccase activity of the transformants was also affected (Figure 1b). The T7 transformant was the least affected, presenting with 2.32 ± 0.1 IU/mg ms, which represents 98% of activity, without differences relative to the PoB (*p* ≤ 0.05). However, the greatest decrease in enzymatic activity was found in strains T21 (1.25 ± 0.18 IU/mg ms), T26 (0.96 ± 0.05 IU/mg ms), and T27 (0.68 ± 0.18 IU/mg ms) corresponding to 55.5, 42.6, and 30.22%, respectively. These activities were significantly different (*p* = 0.01) than those registered for the PoB strain (2.25 ± 0.15 IU/mg ms).

The mycelium developed in these plates served as inoculum for the solid cultures, for which wheat straw was used as a support. PoB (wild type) and all transformants were grown on straw for 38 days, and the mycelium that formed was used to determine the expression of the *lacc2* gene transcripts (Figure 1c).

Measurement of the transcripts in the transformants revealed different levels of *lacc2* expression. At T7, only 2.4% of its expression was inhibited, without significant differences relative to the PoB (F_α(4,10)_ = 4.87; *p* ≤ 0.05). In contrast, the attenuation in the other transformants was 53% for T21 and 61% and 70% for T26 and T27, respectively (Figure 1c). The value of the wild strain was taken as 100% expression.

Taken together, these data confirm that the *lacc2* gene was attenuated in the transformants, and consequently, their laccase activity reduced, with implications for the growth of the transformants.

### 2.2. Expression of the lacc2 Gene in Different Structures of P. ostreatus and Transformant (T7)

**Figure 2:**
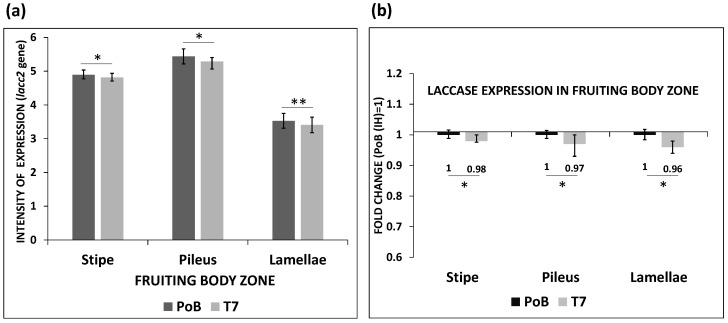
Evaluation of transcripts in different areas of the fruiting body. (**a**) Expression profiles recorded in the stipe, pileus, and lamellae of the PoB and transformant T7 strains. (**b**) mRNA fold change between the strains caused by silencing. Asterisks indicate values with statistically significant differences determined with one-way ANOVA, followed by Tukey’s test (* *p* ≤ 0.05, ** *p* ≤ 0.01).

The expression of *lacc2* was also measured in different regions of the fruiting bodies formed in the PoB and T7 strains (Figure 2). The results showed that the expression in the stipe, pileus, and lamella did not differ between these strains (F_α(1,4)_ = 7.7; *p* ≤ 0.05). The highest levels of expression were found in the pileus, followed by those in the stipe and, finally, those in the lamellae (Figure 2a); the values of expression in the lamella significantly differed from those in the other structures according to the ANOVA analysis and Tukey’s test (F_α(1,4)_ = 5.14; *p* ≤ 0.05).

In addition, the values of the transcripts obtained from the T7 transformant indicated that the inhibition of *lacc2* in the stipe (2%), crown (3%), and laminas (4%) was similar to that found in the mycelium. The expression between strains did not show differences according to Tukey’s test (Figure 2b).

### 2.3. Growth Analysis of P. ostreatus and Transformants in Solid Culture

#### 2.3.1. Effect on Mycelium

**Figure 3:**
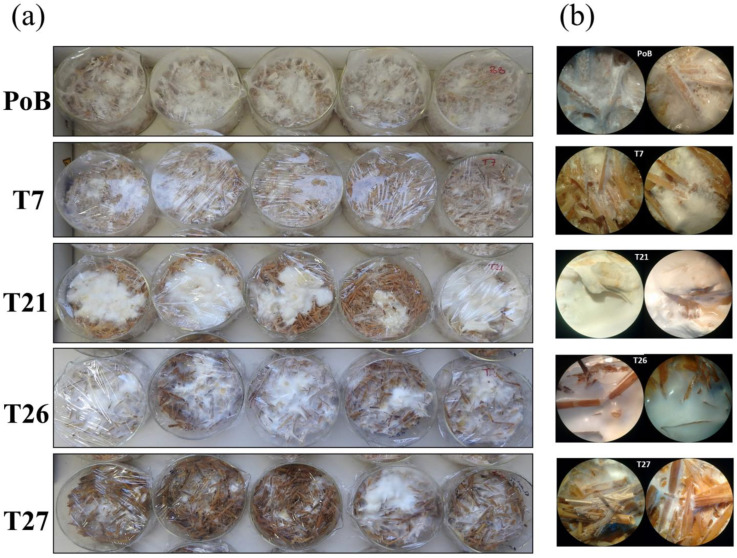
Growth of *P. ostreatus* and silenced strains in solid culture. (**a**) Mycelium developed from PoB, T7, T21, T26, and T27 strains grown on wheat straw. (**b**) Straw samples with cultured mycelium of each strain viewed with a stereomicroscope; Magnification 32×.

In order to compare the development of the PoB strain with that of the transformants, all strains were grown on wheat straw using two sizes of inoculum. Details of solid cultures are described in Section 4.2. From the stage of preparation of the primary inoculum (growth on wheat seeds) and its subsequent cultivation on straw, differences in the colonization capacity between strains were observed. The PoB strain and the T7 transformant were the first to colonize the wheat straw after 17 days of culture, with dense mycelium in both strains (Figure 3a). Unlike these strains, in the T21, T26, and T27 transformants, the homogeneous colonization of the mycelium on the substrate was not observed, instead forming agglomerates (Figure 3a). Furthermore, observations of straw samples from the T26 and T27 crops by dissection microscopy showed dispersed mycelium, lax consistency, and loss of aerial mycelium (Figure 3b).

#### 2.3.2. Effect on the Formation of Primordia and Fruiting Bodies

**Figure 4:**
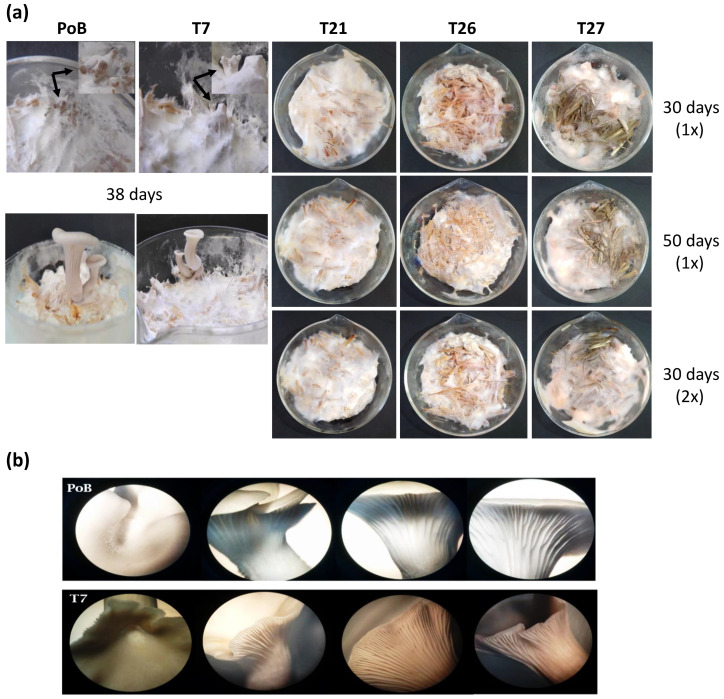
Cultures on wheat straw of the wild-type (PoB) and transformant (T7) strains using two inoculum sizes and incubation times. (**a**) Mycelial density and development of primordia and fruiting bodies. (**b**) Comparison of fruiting bodies obtained between strains after 38 days of culture; Magnification 32×.

After 30 days of culture (Figure 4a), the strains were observed to have fully colonized the straw. The glucosamine method was used to estimate the growth of the strains. The PoB and T7 strains registered the highest amount of biomass (16.62 ± 1.6 mg/g ms) of the cultures, followed by T21 and T26 (13.34 ± 0.09 and 12.46 ± 0.5, mg/g ms, respectively). The strain that generated the least biomass was T27 (10.43 ± 0.26, mg/g ms). The produced biomass data are shown in Supplementary Figure S1a. At day 50, the cultures of the transformants had not produced a biomass equivalent to that reported for the PoB strain; the final biomass obtained in the T21, T26, and T27 strains was 16.3, 15.51, and 14.28, respectively (Supplementary Figure S1b). Even those cultures with double inoculum (2× and incubated for 30 days did not improve biomass production, with growth was similar to cultures with 1× inoculum of the PoB and T7 strains (Supplementary Figure S1c).

Another observation of the cultures was the formation of primordia. In cultures with 1× inoculum and incubated for 30 days, the primordia were visualized only in the wild-type strain and the T7 transformant (Figure 4a, indicated with arrows). The rest of the transformants (T21, T26 and T27), including the cultures incubated for 50 days and those that had twice the inoculum (2×), did not present primordia formation (Figure 4a). In addition, the mycelium developed in the PoB and T7 strains was abundant, with homogeneous colonization, unlike the mycelium formed in the T21, T26, and T27 transformants.

Fruiting bodies were only produced in the PoB and T7 strains, with average formation between strains of approximately 38 days (Figure 4a). The harvest was performed when the fruiting bodies were mature and fully extended. The obtained pilei were funnel-shaped and whitish in color; the blades were decurrent and thin, slightly tight with lamellae (Figure 4b). Although the T7 strain presented low silencing of the *lacc2* gene (2.4%), its moderate differences were determined relative to the fruiting bodies of the PoB strain, such as fruiting times (at least three days apart), change in color and morphology (Figure 4b), and other parameters that are detailed in Table 1. However, determined values such as days of fruiting, stipe measurements, and pileus and fresh weight, as well as the statistical analysis carried out by Student’s *t*-test, indicated that there were no significant differences between the fruiting bodies formed from the PoB and T7 strains.

Interestingly, the transformants (T21, T26, and T27) with more than 50% attenuation of *lacc2* did not have the ability to form fruiting bodies (Figure 4a), despite their longer incubation time (50 days) and the use of double inoculum (2×). These results suggest that *lacc2* participates in the morphogenesis process of *P. ostreatus* because the inhibition of the gene influences its enzymatic activity, consequently affecting the development of the mycelium, and prevents it from completing its biological cycle. According to these results, the changes in the morphogenesis of the silenced *P. ostreatus* strains may be caused by the condition of the hyphal cell walls, whereby the lack of laccase affects the synthesis of β-1,3-1,6-glucan, as well as the thickness and strength of the hyphae, as experimentally demonstrated by Sakamoto et al. [20].

### 2.4. Effect of Laccase Gene Silencing on Micro- and Ultrastructure of Fruiting Bodies

**Figure 5:**
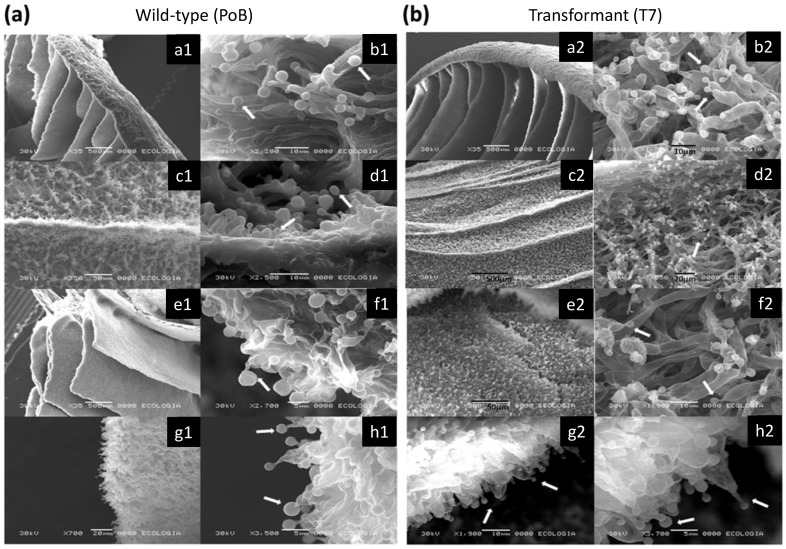
Scanning electron microscopic (SEM) images of the fruiting body of the (**a**) PoB (a1–h1) and (**b**) T7 (a2–h2) strains. (**a**) Pileus, (**b**) pileocystidium, (**c**) stipe, (**d**) caulocystidium, (**e**) hymenium, (**f**) pleurocystidium, (**g**) edge of the hymenium, (**h**) cheilocystidium. The arrows indicate cystidia.

Scanning electron microscopy (SEM) observations were performed on specific areas of the fruiting bodies of the wild-type (PoB) strain and the T7 transformant (Figure 5a,b, respectively). Observations of the pileus, stipe, and hymenium (Figure 5, panels a1,a2, c1,c2 and e1,e2, respectively), did not show morphological changes between the PoB and T7 strains. In addition, Figure 5 shows other analyzed regions of the fruiting bodies such as pileocystidia (b1,b2), caulocystidia (d1,d2), pleurocystidia (f1,f2), and cheilocystidia (h1,h2); according to the obtained images, these structures were similar between strains. SEM imaging of PoB and T7 strain samples, captured structures in the form of ovoid cystidia with a capitate apex in great abundance (Figure 5, indicated with arrows). These structures are characteristic of the genus *Pleurotus* [25]. In each of the analyzed regions of the fruiting bodies, no morphological changes were detected between the PoB and T7 strain, which was expected, as this transformant maintained *lacc2* expression, and its enzymatic activity was similar to the wildtype.

### 2.5. Effect of Laccase Gene Silencing on Mycelial Ultrastructure

Species such as *P. ostreatus*, *P. florida*, *P. eryngii*, *P. cornucopiae*, *P. sajor-caju* [25], *P. cystidiosus* [26], and *P. ostreatus* strain PC9 [27] have been documented to produce specialized structures called toxocysts, which are formed on specific areas of the hypha. The toxocysts described in these reports consisted of a fine, stipe-like sterigma (1.5–4 µm in length) protruding from the aerial hyphae and an ovoid, knob-like structure (2.5–3.5 µm in width) [26]. These descriptions largely coincide with the structures observed in this study.

**Figure 6:**
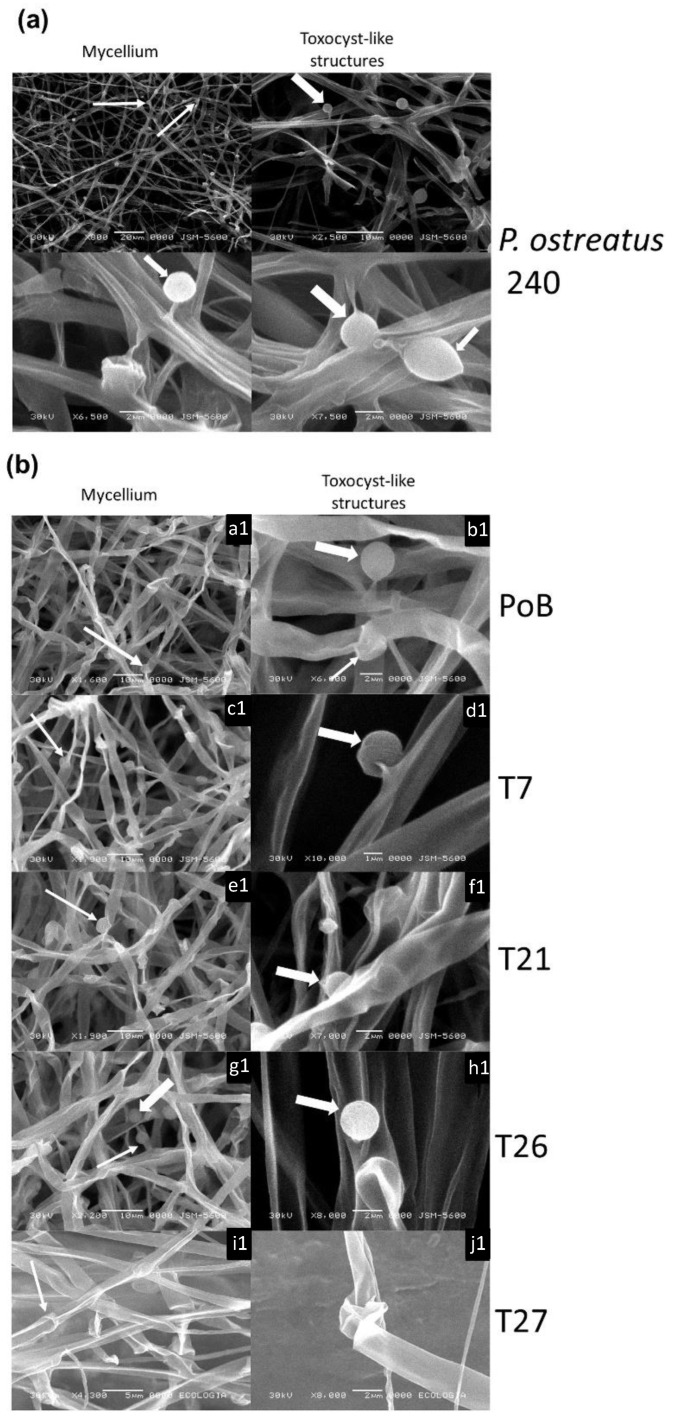
Images of the mycelium developed in MEA medium after 15 days of culture and obtained by scanning electron microscopy (SEM). (**a**) Images of the mycelium and toxocyst formed in the *P. ostreatus* 240 control strain and (**b**) PoB (**a1**,**b1**), T7 (**c1**,**d1**), T21 (**e1**,**f1**), T26 (**g1**,**h1**), and T27 (**i1**,**j1**) grown under the same conditions. The thick arrows indicate some toxocyst-like structures.

To confirm the presence of toxocysts in this work, the wild-strain (PoB) and all the transformants were grown in Petri dishes with MEA medium. For this experiment, the *P. ostreatus* 240 strain was used as a control. This strain is known to produce toxocysts, which allowed us to compare and validate the obtained results. All strains were incubated for approximately 15 days until complete development. SEM images showed the formation of toxocysts in the reference strain (Figure 6a). In the wild strain (PoB) and transformant T7, the same type of ovoid structures were visualized, which were considered toxocyst-like structures (Figure 6, images a1–d1). The abundance of these structures was higher in these two strains compared to that observed in the other strains. In the mycelium of transforming strain T21, a decrease in the abundance of these structures could be observed (Figure 6e1,f1). In the T26 transformant, after careful inspection, only a toxocyst-like structure was observed (Figure 6h1), whereas in the T27 transformant, no such structures were found (Figure 6i1,j1). The results suggest that the toxocyst-like structures are typical of members of this genus and do not represent any contamination of the strains or cultures.

**Figure 7:**
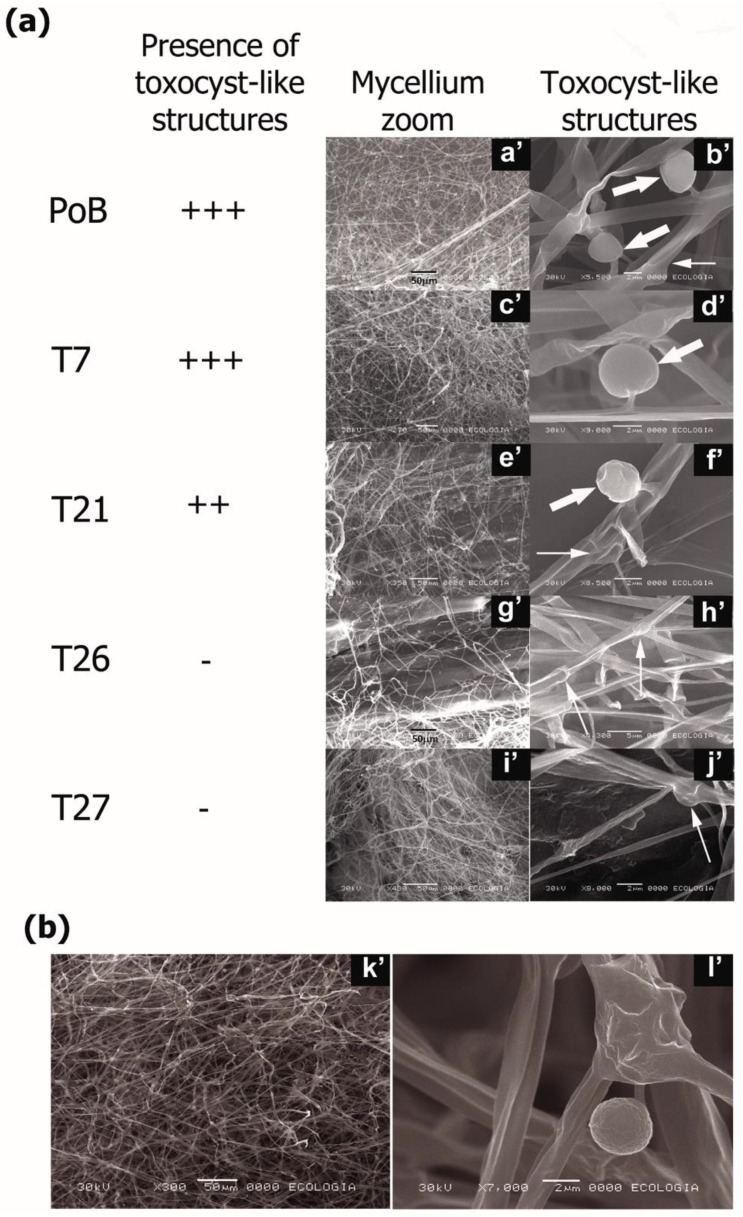
Images obtained from mycelium grown on wheat straw by scanning electron microscopy (SEM). (**a**) Images of the colonized mycelium (**a’**,**c’**,**e’**,**g’**,**i’**) and of toxocyst-like structures (**b’**,**d’**,**f’**,**h’**,**j’**) in PoB, T7, T21, T26, and T27 strains (**b’**,**d’**,**f’**,**h’**,**j’**). (**b**) Close-up images of the mycelium (**k’**) and toxocyst (**l’**) of *P. pulmonarius* IE-115 used as control and grown under the same conditions. Thick arrows indicate toxocyst-like structures and thin arrows point to fibulae.

In the same way, samples were obtained from the solid cultures grown on wheat straw after 38 days and were analyzed by SEM. This analysis revealed ultrastructural differences and the presence of structures such as capitate cystidia (toxocyst-like structures) in the mycelium of the PoB strain and only in the T7 and T21 transformants (Figure 7a). The first column in Figure 7 shows a close-up view of the mycelium of the strains (Figure 7, panels a’,c’,g’,e’,i’). The second column shows structures similar to toxocysts present in the PoB, T7, and T21 strains (Figure 7, panels b’,d’,f’, respectively) and absent in the T26 and T27 strains (Figure 7h’,j’, respectively).

According to the observations of the mycelium, structures such as septa, ramifications, and fibula (Figure 7a, panels b’,f’,h’,j’, indicated with thin arrows) did not differ between the strains. However, the presence and abundance of toxocysts was limited in the T21, T26 and T27 strains (Figure 7a, panels f’,h’,j’) compared to the wild-type and T7 strains.

To corroborate the results and rule out possible contamination, the mycelium of the IE-11 strain of *P. pulmonarius* developed on wheat straw for 38 days was used as an external control. This mycelium also presented toxocyst-like structures (Figure 7b, panel l’), similarly to those observed in the wild strain (PoB) and transformants (T7 and T21).

The tendency to decrease the toxocyst-like structures (Figure 7a), which was directly proportional to the level of affectation of the transformants, was also correlated with the capacity of the strains to form fruiting bodies. Changes in the mycelium produced, null formation of primordia and fruiting bodies, and the absence of toxocyst-like structures confirm that *lacc2* plays an essential role in the morphogenesis process of *P. ostreatus*. This was corroborated by the transformants with medium (T21) and severe (T26 and 27) involvement, which presented a decrease in laccase activity (≤55.5%).

## 3. Discussion

*Pleurotus ostreatus* is a white rot fungus that belongs to basidiomycetes (order Agaricales) and is characterized by degradation of plant components such as cellulose, hemicellulose, and lignin [7]. This property is attributed to lignin-modifying enzymes, including laccase (Lacc) [9]. This enzyme, in addition to processing lignin, has the ability to oxidize different phenolic and non-phenolic substances, with the particularity of only using molecular oxygen for its reaction [10,11,12]. However, the activity of fungal laccases is not limited to lignin degradation; their participation in biological functions such as virulence, defense/protection, pathogenesis, pigmentation, and sporulation processes has also been reported [14], which justifies the relevance of the analysis of laccases produced in different classes of fungi belonging to Deuteromycetes, Ascomycetes, and Basidiomycetes [28]. In this context, the purification of these enzymes has contributed to understanding of biochemical properties [29] and gene regulation studies have elucidated some of their physiological implications [14,30]. Owing to their versatility, these enzymes have also been proposed for biotechnological use in the textile, paper, and food industries [8,9,11].

On the other hand, the sequencing of some genomes has revealed the number of genes that code for laccases, *P. ostreatus* DSM11191 (12), *P. ostreatus* PC9 (11), and *P. ostreatus* PC15 (14) (https://mycocosm.jgi.doe.gov/mycocosm/home/releases?flt=Pleurotus+ostreatus, accessed on 28 February 2023). Several studies have reported that the expression of these genes is differential [31] and may depend on factors such as the composition of the medium and the presence of metal ions (Cu^2+^, Mn^2+^) [32,33] and phenolic compounds [34]. Castanera et al. [16] showed that in *P. ostreatus* under liquid and solid culture conditions (induced by Cu^2+^), only two laccases (*lacc2* and *lacc10*) were consistently expressed over time. The *P. ostreatus* PoB strain has been cultivated in liquid and solid media, and both conditions have been observed to consistently produce an extracellular laccase of approximately 37 kDa [24,35]. Through expression analysis of the PoB strain from liquid and solid cultures, Armas-Tizapantzi et al. [24] found that the *lacc2* and *lacc10* transcripts had the highest expression, as similarly reported by Castanera et al. [16].

Despite these antecedents, studies on the regulation of the genes that encode laccases in *P*. *ostreatus* have not been previously reported, emphasizing the importance of having obtained transformants with different levels of *lacc2* expression such as T7 (97.6%), T21 (47%), and T26 and 27 (39% and 30%, respectively). These strains were cultivated in solid medium on wheat straw to analyze their possible effects on the morphogenesis of the fungus at the macro- and micro morphological level.

In these cultures, we observed that the growth was similar between the wild-type and T7 strains up to 38 days. However, transformants with more than 50% attenuation of *lacc2* showed differences such as speed of colonization of the substrate, lower mycelium density, random agglomerations, and the absence of aerial mycelium (Figure 3). However, the most relevant effect obtained in strains T21, T26, and T27 was the fact that they did not develop primordia and fruiting bodies, despite having been cultivated for longer (50 day) and with the use of a double inoculum (Figure 4). This may have been caused by the remodeling of the hyphal cell wall components (decrease in glucans), as demonstrated in *Lentinula edodes* [20].

Previous studies reported the possible functions of laccases and their regulation in their different stages of development in some fungi, which are in agreement with our results. In *L. edodes*, the increase in laccase activity was observed to be associated with the formation of aerial mycelium, primordia, and fruiting bodies [36]. The same effect was confirmed by overexpression of the *lacc1* gene in *Hysizygus marmoreus* [37].

In contrast, when the expression of genes coding for laccases is inhibited by a methodological strategy, important effects on development and/or fungal structures have been observed. Das et al. [38] generated mutants of *P. florida* by exposure to *N*-Methyl-*N*′-Nitrosoguanidine, which presented low LACC production, in addition to showing difficulty in developing mycelium and not forming fruiting bodies. Recently, Cesur et al. [39] reported the direct participation of extracellular laccases in the development of fruiting bodies when comparing degenerate strains of laccases against the wild-type strain of the fungus *Flammulin velutipes*.

Furthermore, the regulation of the gene that codes for LACC1 has been analyzed in different species of fungi, with the results demonstrating important effects on morphogenesis in each of them. In this sense, Nakade et al. [19] reported that attenuation by gene silencing of *lacc1* in *L. edodes* caused changes in the morphology of the mycelium (low density and thin cell wall), while in the fungus *H. marmoreus*, *lacc1* silencing affected the formation of mycelium, primordia, and fruiting bodies [37]. Mutants of the *Ganoderma tsugae lacc1* gene showed clear effects on the pigmentation and morphology of their fruiting bodies, which were smaller with respect to the control strain [21]. However, one such study that can be correlated with the present results was carried out by Sakamoto et al. [20], who silenced the *lacc1* gene of *L. edodes*. One of the transformants (ivrL1#32) presented abnormal hyphae and less aerial mycelium and did not form fruiting bodies. In addition, the mycelium showed a thinner cell wall, due to a lower content of β-1,3-1,6-glucan. These authors demonstrated that the composition of the cell wall in ivrL1#32 was reversed by the addition of purified laccase to the culture medium.

Nevertheless, few studies have described the physiological role of LACC2. Jiao et al. [13] reported on the analysis of the genes that encode LACC2 and LACC12 in *P. ostreatus* and found that these enzymes are related to lignin degradation and fruiting body formation, respectively. With the overexpression of the *lacc2* gene, they observed that lignin degradation was greater in the recombinant strains than in the wild strain. Recently, nutritional factors were reported to be essential for the production of *P. ostreatus* laccases in submerged fermentations and respective transcriptomic analyses showed that presence of copper induces the expression of the *lacc2*, *lacc6*, and *lacc10* genes [40].

This constant and high expression of *lacc2* found in both liquid and solid culture and reported by several authors [17,24,31,32,35,40] shows its relevance in *P. ostreatus*. According to our results, the LACC2 enzyme seems to play an essential role in the morphology of the fungus as its inhibition prevents the formation of micro and macroscopic structures.

The expression levels of *lacc2* were also measured in different regions of the fruiting bodies formed by the wild-type and transformant T7 strains. The *lacc2* transcripts between the strains did not show differences, although the highest levels of expression were recorded in the pileus (5.4 ± 0.219) and stipe (4.9 ± 0.12), compared to those determined in the lamellae where the expression decreased markedly (3.5 ± 0.22). The low levels of the transcripts in the lamellae are possibly due to the fact that the *lacc2* gene is regulated by an endogenous compound of the fungus. Laccase genes are known to be regulated by environmental factors, metal ions, or phenolic compounds [41], which has been confirmed by transcriptomic and proteomic studies in *P. ostreatus* [42]. In this sense, Chakraborty et al. [43] also observed a decrease in the laccase activity of *P. florida* in the lamellae during sporulation, attributing this decrease to the presence of an endogenous substrate that inhibits the laccase activity accumulated in these structures.

Finally, SEM was used to detect toxocyst-like structures in the strains analyzed in this work as previously reported by Armas-Tizapantzi et al. [44]. Interestingly, the presence of toxocysts was found to depend on silencing, because in the strains with the highest *lacc2* attenuation, these structures decreased drastically (T21 and T26) or were not detected (T27) (Figure 7). As mentioned, even these strains did not produce fruiting bodies, which suggests that *lacc2* is required for the formation of toxocysts; in turn, these structures are directly related to the development of fruiting bodies. On the other hand, Truong et al. [26] documented that some *Pleurotus* have the ability to parasitize nematodes, which was later shown to be due to the presence of toxocysts. *P. ostreatus* toxocysts were determined to contain trans-2-decanoic acid, a compound that exhibits adhesion properties and toxicity against nematodes [45]. Another speculation is that the elastic envelope of the toxocyst balloon is composed of a substance similar to latex [26], which also confers the property of adhesion on nematodes. A new report on *P. ostreatus* indicated that the toxocysts of the fungus contain a volatile ketone, 3-octanone, a molecule biotoxic that can damage the integrity of the cell membrane, which facilitates the entry of extracellular calcium into the cytosol and the mitochondria causing the death of the nematodes [27]. Thus, the silenced strains can offer new perspectives for studies, providing additional evidence of these phenomena and supporting future work in relation to the presence and absence of toxocysts with respect to their biological application to different pathogens.

## 4. Materials and Methods

### 4.1. Fungal Strains

The *P. ostreatus* PoB (wild strain) was obtained from the collection of the Laboratorio Institucional de Hongos Comestibles de la Universidad Autónoma Chapingo (Texcoco, Estado de México, México). This strain produces only one constant laccase isoform under the conditions of culture tested here. The transformant strains T7, T21, T26, and T27 (silenced for *lacc2*) were obtained by the RNAi mechanism using the *P*. *ostreatus* PoB strain as described by Armas-Tizapantzi et al. [24]. Transformant strains were obtained using the plasmid pRNAi-LAC, which contains the promotors of glyceraldehyde-3-phosphate dehydrogenase (*gpd*) of *Aspegillus nidulans* and pyruvate kinase (*pki*) genes of *A. niger*. These promoters were placed in opposite directions, one in front of the other, and complementary RNA molecules were formed through the transcription of each of them, generating double-stranded RNA (dsRNA). The DNA fragment of the *lacc2* gene used to generate the dsRNAs was 317 bp.

The *P. pulmonarius* IE-11 and *P. ostreatus* 240 strains were donated by the Instituto de Ecología A.C. (Xalapa, Veracruz). These strains were used as reference to rule out contamination and validate the structures formed in *P. ostreatus* (PoB) and transformants.

### 4.2. Culture Conditions

*P. ostreatus* (PoB), *P. pulmonarius* IE-11, and transformant strains were cultivated on wheat straw following the protocol previously reported by Sainos et al. [46] with some modifications. The culture procedure was implemented in the following order.

Propagation of mycelium: Petri dishes containing potato dextrose agar (PDA) or malt extract agar (MEA) were used to propagate the mycelium of all strains.

Primary inoculum: Wheat seeds were used as the substrate. The seeds were washed, immersed in water for 12 h, and drained until they reached 70–80% humidity. Next, the wheat seeds (300 g) were placed in polyethylene bags (18 × 25 cm) and autoclaved at 121 °C for 20 min. Then, each bag was individually inoculated with three plugs (0.5 cm^2^) of mycelium (previously propagated in MEA) from each of the transformant strains and the wild strain. The inoculated bags were incubated at 25 °C until complete colonization of the seeds.

Solid culture: The wheat straw used for the culture was cut into pieces approximately 2 to 5 cm long. The straw was soaked for 18 h and drained until it reached 70–80% humidity. Then, 30 g of this wet straw was placed in glass crystallizers (9 cm in diameter × 5 cm in height) and autoclaved at 121 °C for 20 min. After sterilization, wheat straw was inoculated with 35 seeds of the primary inoculum and incubated at 25 °C in the dark, covered with aluminum. Five replicates of the wild strain and the transformants were made.

The cultures were revised after 8 days, allowing the colonization of the mycelium on the straw. Once the mycelium had colonized the entire substrate, the fruiting phase began. The crystallizers were exposed to a natural cycle of light and dark (12 h light/12 h darkness) at room temperature. At this time, the aluminum lid was changed for a plastic Egapack type; the lids were perforated to allow oxygenation, and crystallizers were sprayed every 48 h with 400 µL of sterile distilled water to maintain 80–90% humidity in the cultures. The fungal cultures lasted about 38 days to form the fruiting bodies.

To obtain toxocyst-like structures of *P. ostreatus* PoB and transformants, the strains were cultivated in MEA medium, placing a fragment of agar with mycelium for growth. The plates were incubated at 25 °C for 15 days in darkness. *P. ostreatus* strain 240 was used as a control for this experiment.

### 4.3. Biomass Determination of P. ostreatus PoB and Transformant Strains

The wild-type PoB strain and the transformants (T7, T21, T26, and T27) were grown in crystallizers (as detailed in the previous section). Biomass measurement in the wild-type strain and transformants was performed at 30 and 50 days of culture, using 35 wheat seeds (1× inoculum) because the transformants T21, T26, and T27 showed a delay in the formation of mycelium and primordia at the aforementioned times as a possible cause of silencing. Simultaneously, cultures of these strains were carried out under the same conditions but using 70 seeds of the primary inoculum (2× inoculum) and their biomass was measured at 30 days. After the corresponding incubation period, the crystallizers were placed in an oven at 45 °C for 5 days, and dry samples were weighed and pulverized. Then, 2 g of each culture was used for biomass determination through glucosamine quantification following the protocols of Scotti et al. [47] and Marcial et al. [48]. Standard curves were made with glucosamine (1 mg/mL) and dry biomass of *P. ostreatus* (0–150 mg) to obtain the glucosamine content/biomass ratio. Biomass data were expressed in mg of biomass/g of solid medium (mg/g ms). Three replicates were realized for each strain.

### 4.4. Extracellular Laccase Activity Determination

The laccase activity of *P*. *ostreatus* PoB and transformants was determined from plate cultures using YMG solid medium. After eight days of fungal growth, the enzyme extract was obtained. To this end, the mycelium was separated from the medium and the agar was used to obtain the secreted laccase. The agar present in the Petri dish was transferred to a flask with 15 mL of phosphate buffer (0.5 M, pH 6.5) and shaken for 15 min. Next, the mixture was filtered through filter paper (Whatman No. 1) and the filtration product was used as enzyme extract and stored at –20 °C [24]. Measurement of laccase activity was performed as follows: the reaction mixture (1 mL) contained 2,6-dimethoxyphenol (2 mM) in potassium phosphate buffer (0.1 M, pH 6.5) and 200 µL of enzyme extract. The mixture was incubated for 4 min at 39 °C, and then measured at 468 nm in a SmartSpec Plus spectrophotometer (BioRad, Hercules, CA, USA). The enzymatic activity was expressed as international units (IU), where 1 IU is the amount of enzyme that catalyzes the transformation of 1 µmol of the substrate into a product per minute.

### 4.5. RNA Extraction

Total RNA of all the strains was extracted from mycelium grown on wheat straw (38 days of culture). Extractions were performed using a RNeasy Plant Mini Kit (Qiagen, Hilden, Germany) following the manufacturer’s protocol. The residual DNA from the RNA extraction was removed with DNase I (Thermo Scientific, Inc., Waltham, MA, USA). The integrity of the RNA samples was verified through ethidium bromide-stained agarose gels (1%), and the concentration of RNA was measured using a SmartSpect Plus spectrophotometer (BioRad, Hercules, CA, USA).

### 4.6. cDNA Synthesis and PCR

cDNA synthesis was performed from 3 µg of total RNA (previously treated with DNase I) as a template using a RevertAid H Minus First Strand cDNA Synthesis Kit (Thermo Scientific, Inc., Waltham, MA, USA) according to the conditions established in the manual. Then, the *lacc2* and actin gene fragments were amplified by PCR. The reaction mixture contained cDNA (20 ng), dNTPs (10 mM), buffer (10X) with MgCl_2_ (2 mM), primers (0.4 µM), and Taq DNA polymerase (0.1 U) (Thermo Scientific, Inc., Waltham, MA, USA). The primer pairs used to evaluate the expression of the laccase gene were those corresponding to the *lacc2* (fw 5′-GAAGCTGGTCTCGCTGTTGTC-3′ and rev 5′-GTTCGCCCTCGTTGACTTCA-3′) and actin genes (fw 5′-TCCCTCAGCACCTTCCAGAA-3′ and rev 5′-GGGCCGGACTCGTCGTA-3′); the latter was used as an endogenous expression control. The fragments obtained with theses primers were 100 pb, similar to those previously reported by Pezella et al. [17].

PCR was performed in a T100 Thermal Cycler (BioRad, Hercules, CA, USA) under the following conditions: 95 °C for 4 min, followed by 30 cycles at 95 °C for 1 min, 55 °C for 1 min, 72 °C for 1 min, and a final cycle of 72 °C for 7 min. The results of the amplifications were loaded and run on 2.0% agarose gels using TAE 1X and stained in an ethidium bromide solution. Gels were visualized on a DigiDoc-it photo documenter (UVP, Jena, Germany). The normalized intensity of the amplicons was obtained using ImageJ software (version 153K, Bethesda, MD, USA), which is a digital image processing program developed by the National Institutes of Health. With this software, data of relative intensity were obtained. By dividing the relative intensity of each mRNA and each strain by the respective relative intensity of actin, the value of the normalized intensity was obtained.

### 4.7. Macro- and Microscopic Morphology Evaluation

The times of substrate invasion and development of primordia were recorded. The conventional morphology characteristics such as fruiting body size, color, and diameter of the pileus were measured. The fruiting bodies were harvested when the pileus was fully extended, and were weighed fresh. Furthermore, the diameter of the hyphae and the presence/absence of fibulae and basidia were recorded. Mycelium and fruiting bodies developed on wheat straw were observed with a Stemi DV4 binocular stereomicroscope (ZEISS^®^, Göttingen, Germany).

The samples of wheat straw invaded with mycelium and the fruiting bodies, as well as the samples of mycelium after 15 days of development on MEA, were observed with a scanning electron microscope (JEOL-JSM 5600 LV), in the Electron Microscopy Laboratory at the Instituto de Ecología AC, Xalapa, Mexico. All samples were lyophilized under vacuum, placed on a fixed support, and coated with gold-palladium as a conductive coating (Denton Vacuum DESK V, Denton Vacuum, Inc., Moorestown, NJ, USA). Preparations obtained in this way were analyzed by SEM at different magnifications, obtaining the corresponding digital photographs. For comparation and validation of the results of the mycelium samples in Petri dishes, the *P. ostreatus* 240 strain was used as a reference.

### 4.8. Statistical Analysis

The parameters estimated during the evaluation of the silencing effect of the macro and microscopic morphology data were analyzed using a one-way ANOVA (α = 0.05), and differences between the PoB strain and transformants were assessed using Tukey’s test (*p* ≤ 0.05). Student’s test (*t*-test) was used to compare the different parameters measured in the fruiting bodies formed in the PoB and T7 strains. Statistics were analyzed with IBM SPSS Statistics 25 software.

## 5. Conclusions

In this study, the efficiency of the RNAi technique to silence the *lacc2* gene of *P. ostreatus* was verified. Silencing decreased the laccase activity of the transformants obtained above 50%, which correlated with the macro- and micro-morphological changes of these strains. Through solid cultures, we demonstrated that transformant strains with downregulation of the *lacc2* gene did not form aerial mycelium, primordia, or fruiting bodies. Electron microscopy showed evidence of ovoid formations adhered to the mycelium that were identified as toxocyst-like structures, which enabled the determination of, according to the degree of *lacc2* attenuation in the transformants, their presence and abundance. In this sense, the results suggested that *lacc2* is required for the formation of toxocysts and that the absence of these structures is a necessary condition for the development of primordia and fruiting bodies of the fungus.

Finally, the approach described in this study can also allow us to analyze the expression of other *lacc* genes to elucidate their possible functions in the morphogenesis of this fungus. Moreover, these transformants can be used to examine their participation in lignin processing and their ability to degrade toxic compounds or analyze antagonistic properties against pathogenic fungi, as well as nematicidal action.

## Figures and Tables

**Table 1:** Data from cultivation time until harvest and morphological measurements of the fruiting bodies produced by the PoB and T7 strains.

Strain	Days to Fruiting	Stipe Diameter (mm)	Stipe Height (mm)	Pileus Width (mm)	Pileus Length (mm)	Fresh Weight (g)
PoB	36.6 ± 0.89 ^a^	9.022 ± 1.11 ^a^	75.82 ± 6.16 ^a^	27.58 ± 3.56 ^a^	26.33 ±6.01 ^a^	4.4146 ± 1.26 ^a^
T7	39 ± 4.6 ^a^	8.134 ± 0.91 ^a^	64.3 ± 9.79 ^a^	26.69 ± 3.34 ^a^	24.8 ± 4.38 ^a^	3.4921 ± 0.88 ^a^
	*p* = 0.294	*p* = 0.206	*p* = 0.057	*p* = 0.692	*p* = 0.660	*p* = 0.219

Statistical analysis by Student’s test (*t*-test). Superscript letters indicate statistical comparisons between strains. Different letters denote statistically significant differences (*p* ≤ 0.05).

## Data Availability

Not applicable.

## Supplementary Materials

The following supporting information can be downloaded at: https://www.mdpi.com/article/10.3390/ijms24098143/s1.
