# Supplementary material for: Silencing of the Laccase (lacc2) Gene from Pleurotus ostreatus Causes Important Effects on the Formation of Toxocyst-like Structures and Fruiting Body
Source: Int J Mol Sci. 2023 May 2;24(9):8143. doi: 10.3390/ijms24098143 (PMC10179115; doi:10.3390/ijms24098143)
Supplement: Supplementary file 1 [file ijms-24-08143-s001.zip › ijms-2342834-supplementary.pdf]

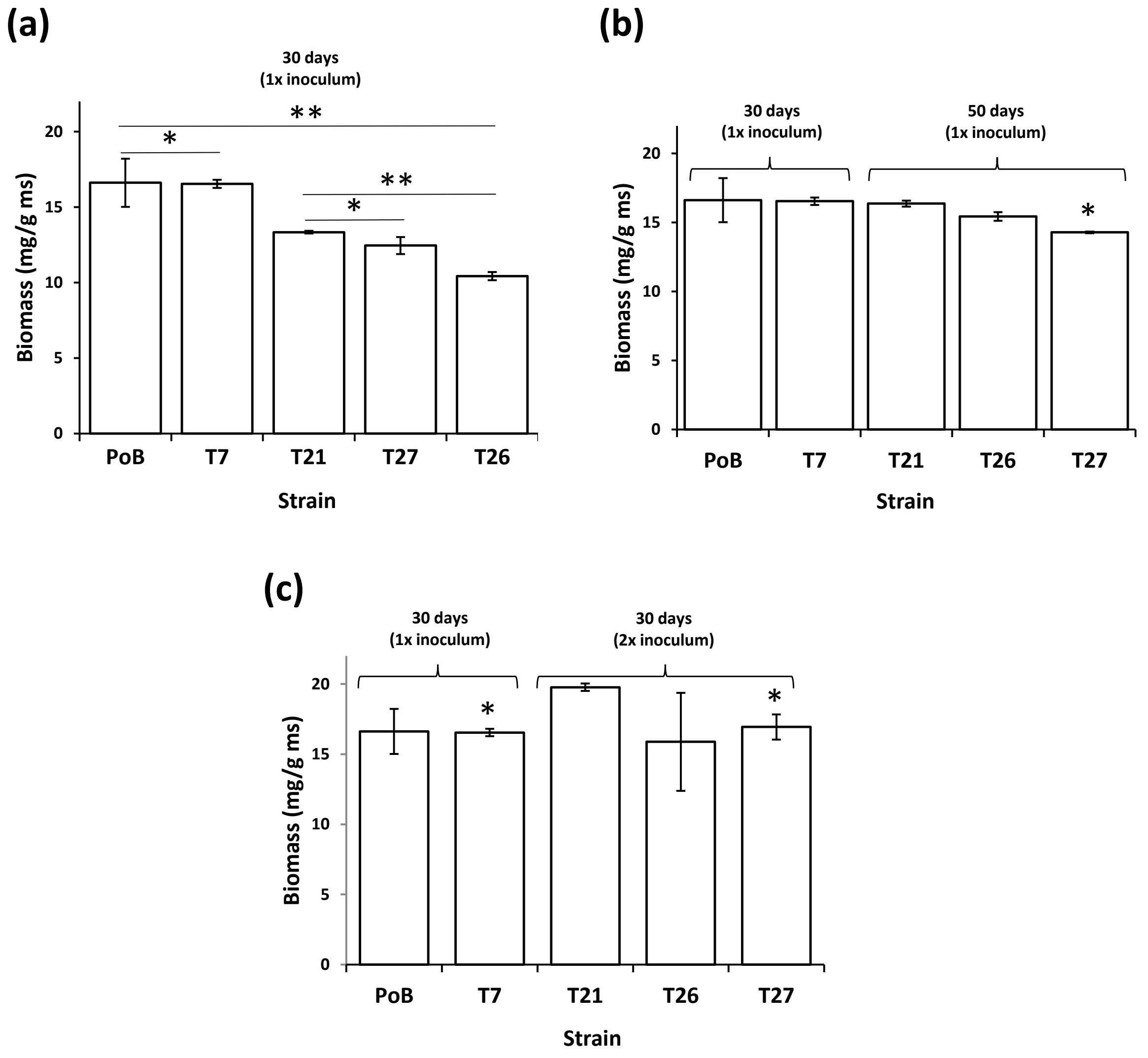

Supplementary Figure S1. Growth of the wild strain PoB and transformants (T7, T21, T26, and T27). (a) Biomass measurement by the glucosamine method at 30 (a) and (b) 50 days of culture and normal inoculum (1x). (c) Quantification of biomass at 30 days of culture and with double inoculum. The experiments were performed in triplicate and the error bars represent the standard deviation. * Indicates significant differences with respect to the wild strain (*$p \leq 0.05$ and **$p \leq 0.001$).
